# Polyethyleneimine-mediated gene transfection in porcine fetal fibroblasts

**DOI:** 10.1590/1984-3143-AR2024-0026

**Published:** 2024-11-22

**Authors:** Andressa Pereira de Souza, Ana Paula Bastos, Francisco Noé da Fonseca, José Rodrigo Pandolfi, Carlos André da Veiga Lima Rosa Costamilan, Mariana Groke Marques

**Affiliations:** 1 Centro de Ciências Agroveterinárias, Universidade do Estado de Santa Catarina, Lages, SC, Brasil; 2 Empresa Brasileira de Pesquisa Agropecuária, Embrapa Suínos e Aves, Concórdia, SC, Brasil; 3 Centro de Educação Superior da Região Sul, Universidade do Estado de Santa Catarina, Laguna, SC, Brasil; 4 Pós-Graduação em Produção e Sanidade Animal, Instituto Federal Catarinense, Concórdia, SC, Brasil

**Keywords:** primary fibroblast, cationic polymer, non-viral vectors, gene delivery, nuclear transfer

## Abstract

Polyethylenimine (PEI) has been explored as an efficient non-viral system for delivering genes to cells; however, there were no protocols for its use in porcine fetal fibroblasts (PFF). Therefore, we compared different concentrations of FITC-PEI (0.625, 1.25, 2.5, 5, 10, 20, 40, or 80 µg/mL) and incubation times (30 min, 1 h, or 2 h). It was observed that the incubation time did not affect the internalization of the PEI-FITC and that 30 min was sufficient to capture the complex. The concentrations higher than 10 µg/mL could reach many marked PFF (>90%). Then, two PEI concentrations were tested, 10 or 40 µg/mL, combined with an N/P of 2 with the pmhyGENIE-5 for 30 min. The percentage of PFF-GFP positive was similar between the PEI concentrations in the evaluation time points (24 h, 48 h, and 72 h). However, 40 µg/mL caused higher membrane damage rates. Thus, it can be concluded that concentrations between 10 – 80 µg/ml of PEI promote high incorporation rates, even in periods as short as 30 minutes. Furthermore, it can be stated that the transfection condition used in Polyplexes 1 (10 µg/mL of PEI and 37.5 µg/mL of pmhyGENIE-5 for 30 min) efficiently produces genetically edited porcine fetal fibroblasts with low cell damage.

## Introduction

Genetically modified swine holds excellent promise in agriculture and biomedicine. Several potential applications of transgenic pigs have been proposed, including genetic research, recombinant proteins, production, and improved productivity traits.

Somatic cell nuclear transfer (SCNT) is currently the most used method for producing genetically modified pigs. SCNT associated with transgenesis combines animal cloning technique with transgenics to create a cloned genetically modified animal. In this case, a previously genetically modified cell is transferred to an enucleated oocyte. Therefore, the donor cell has a fundamental role in SCNT associated with transgenesis, which can significantly impact the procedure's success. In SCNT techniques, porcine fetal fibroblasts (PFF) are typically used as donor cells (Kühholzer et al., 2001; Lee et al., 2003). However, efficient gene transfection in this cell type is limited, decreasing genetically edited swine production.

The most common methods for cell transfection are electroporation (Andreason and Evans, 1989), trans-duction with lentiviral vectors (Hofmann et al., 2004), lipofection (Felgner et al., 1987), and cationic polymer-based transfection (Pham et al., 2006).

Viral vectors achieve an acceptable efficiency in delivering and expressing exogenous genes for PFF (Lungwitz et al., 2005); however, factors such as immunogenic components, small transgene size, and high cost may limit their use (Akinc et al., 2005).

Electroporation is also frequently employed to transfect PFF (Boussif et al., 1995; Zhou et al., 2012). However, efficiency is typically lower. Besides, non-specific damage to cells is another concern that limits them. In addition, specialized equipment is needed, which can be expensive, limiting the usability and accessibility of these techniques (Yamano et al., 2010).

Biocompatible cationic polymers and liposomes have a wide transfection range and relatively low immunogenicity. Commercial products are available and ready to use, with DNA complexation happening by self-assembly, and no specialized devices or skills are required. Although liposomes are widely used and highly efficient, they are expensive commercial reagents (Sharma et al., 2011).

Among cationic polymers, Polyethyleneimine (PEI) is considered one of the most effective transfection agents. PEI is available in various sizes and structures, but 25 kDa branched PEI has proven an efficient, low-cost, practical, and versatile agent for gene delivery; besides, it can be combined with other techniques (Sharma et al., 2011; Souza et al., 2018).

Its ability to transfect is derived from the high density of positive charges attributed to amine groups, which interact electrostatically with nucleic acids negatively charged phosphate backbone (Rémy-Kristensen et al., 2001). These nanosized complexes, called polyplexes, bind to anionic residues on the cell surface and are taken up via endocytosis (Godbey et al., 1999). The abundance of amine groups exerts the so-called “proton-sponge” effect, which can provide additional protection against exogenous DNA degradation due to their ability to avoid trafficking to degradative lysosomes (Tian et al., 2007).

PEI application as a cell transfection reagent was first proposed by Boussif et al. (1995), who demonstrated that PEI could transfer plasmid DNA (pDNA) to newborn mice's brains. Since then, several studies have described PEI as a good transfectant in various cell types, such as imported human cells (HeLa, Hep G2, MCF-7, SMMC-7721, PK-15) and from mice (4T1, NIH/3T3, C2C12, R1); primary cultured cells (fetal bovine fibroblasts, rat bone marrow stromal cells, primary pig liver cells) and even germ cells such as porcine sperm (Hsu and Uludağ, 2012; Souza et al., 2018; Tian et al., 2007; Fischer et al., 1999).

However, there is no standardized protocol for PEI for PFF transfection to date. Primary tissue-derived cells could be more selective toward the physicochemical properties of the complexes, which can define the predominant endocytic uptake pathway and, ultimately, how positions are processed and transported within the intracellular domains. Thus, it is essential to note that transfection procedures need to be optimized for the individual cell line.

Although PEI is a widely studied transfection mediator, many questions remain open, such as its exact mechanism of action, toxicity, and factors that affect transfection efficiency. Several parameters can affect transfection, including incubation time, media, vector type (format and size of exogenous DNA), polymer concentration, and polymer-to-DNA ratio. PEI efficiency depends on the environment and conditions in which complexes are formed. In some cases, complexes formulated under one condition for transfecting a particular cell line may not be optimal for a different one (Sharma et al., 2011). Thus, new standards must be established for each polymer and each type of cell line.

Thus, this study aimed to optimize the concentration and incubation time for gene delivery in primary cultures of swine fetal fibroblasts, using 25 kDa branched Polyethyleneimine, hence facilitating the transfection of primary cultures, high step importance for SCNT protocols.

## Methods

Unless otherwise indicated, all chemicals used in this study were purchased from Sigma-Aldrich Chemicals (St Louis, MO, USA). The bioethics committee of the Embrapa Suínos e Aves and the Internal Biosafety Commission (CIBIO) approved these experiments (protocols 003/2016 and 01/2015, respectively).

### PEI solution

A branched Polyethyleneimine 25 kDa (18 mM) stock solution was prepared by resuspending 16.2 mg of PEI in 20 ml of PBS (pH 7.0). The solution was vortexed vigorously and kept at room temperature for 24 hours before use to ensure complete dissolution.

### Cell culture

**Figure 1:**
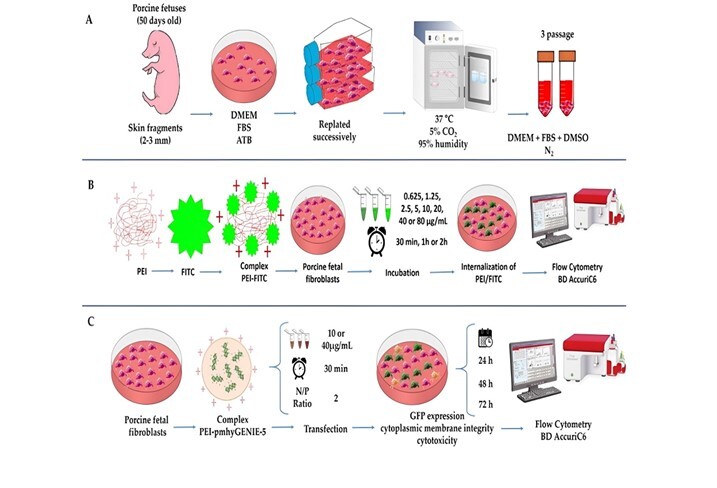
Schematic representation of the experimental design. A. Establishment of porcine fetal fibroblast cell line. B. Experiment 1: Optimization of PEI concentration and incubation time. C. Experiment 2. The efficiency of polyplexes in the production of PFFs expressing GFP. Created in BioRender (Marques, 2024).

PFF cell lines were established from the biopsy of porcine fetuses (approximately 50 days old) obtained in a local slaughterhouse. Skin fragments (2-3 mm) were aseptically obtained and cultured in Petri dishes (35 mm) containing DMEM (DMEM High glucose Gibco®) supplemented with 10% (v/v) fetal bovine serum (FBS - Gibco®) and 50 IU/mL gentamicin. Plated fragments were incubated at 37°C, 5% CO_2_, and high humidity. Upon observing approximately 40% confluence, the fragments were carefully removed. The attached fibroblasts remained in culture until they reached 80-90% confluence when they were trypsinized (trypsin 0.25% with EDTA 0.025% in PBS without calcium and magnesium and replated, successively, until reaching the third passage. Cells were then frozen in a DMEM medium with 20% FBS and 10% dimethylsulfoxide. The fibroblasts were thawed and cultured for the experimental protocols until they reached 80% confluence when they were trypsinized and submitted to the experimental protocols (Figure 1A).

### Experiment 1: Optimization of PEI concentration and incubation time

The PEI was derivatized with a fluorescent probe (FITC), according to Saito and Saitoh (2012), with modifications. Briefly, FITC (10 mM in DMSO) was diluted in PEI solution (20 mg/mL in PBS) to a final concentration of 0.1 mM. The final solution was stirred for 2.5 h at 18°C, followed by dialysis in cold PBS overnight. The final molar proportion of the derivatized micelles was 0.6 mol FITC for 1 mol PEI.

Petri dishes (35 mm) were seeded with 2x10^5^ cells, cultured for 48 h in DMEM + 10% of FBS + 50 IU/mL gentamicin, and incubated under the conditions described earlier. A factorial design (8x3) was used to evaluate the effects of PEI concentration and incubation time. PFFs were exposed to 8 different concentrations of PEI-FITC (0.625, 1.25, 2.5, 5, 10, 20, 40, or 80 µg/mL) for three different incubation times (30 min, 1h, or 2h), performed in quadruplicate. When cells reached 80% confluence, they were washed with DMEM High glucose (without FBS and gentamicin) and subjected to treatment.

To evaluate the percentage of captured PEI-FITC cells, the culture medium was changed for DMEM + 10% of FBS, and cells were removed from the plate with a cell scraper at the end of each period. The cells were analyzed using flow cytometry BD AccuriC6 (Becton & Dickson, Santiago, Chile) equipped with standard optics and an air-cooled argon laser operating at 488 nm excitation and 20 mW, and three-light filters (FL-1: 533 ± 30 nm; FL-2: 585 ± 40 nm; and FL-3: 675 ± 25 nm). Forward scatter (FSC) and side scatter (SSC) were used to gate the PFF population and exclude debris. A total of 10,000 cell events at 600 cells/s were acquired for each measurement. The acquisition was performed with a 14 μL/min sample aspiration speed. Data were acquired on a logarithmic scale. When required, the spectral overlap of each particular staining was compensated. The cells marked with FITC were evaluated in the FL1 channel (FL1: 533 ± 30 nm). All experiments were performed in quadruplicate. The experimental design is shown in Figure 1B.

### Experiment 2: Efficiency of polyplexes in the production of PFFs expressing GFP

#### Preparation of polyplexes

Polyplexes were prepared based on the results of the internalization experiment (PEI: 10 and 40 µg/mL, and incubation time: 30 min). A plasmid was used as a PiggyBac transposase-based integration vector (pmhyGENIE-5) encoding the improved green fluorescent protein (eGFP).

The pmhyGENIE-5 was first complexed to prepare the polyplex with the PEI solution described in item 2.1. After 10 minutes, DMEM (without FBS and gentamicin) was added at the 2 N/P ratio, but altering the concentration as follows: polyplex 1 (10 µg/mL of PEI and 37.5 µg/mL of pmhyGENIE-5) and polyplex 2 (40 µg/mL of PEI and 150 µg/mL of pmhyGENIE-5). In addition, the polyplexes were analyzed in terms of size and zeta potential by dynamic light scattering and electrophoretic mobility using a Zetasizer device (Malvern, UK).

### Evaluation of GFP expression and cytoplasmic membrane integrity

After thawing, fibroblasts were grown in 25 cm^2^ bottles containing complete DMEM for approximately 48 h. Upon reaching 80% confluence, PFFs were treated with trypsin (as previously described), and 2x10^5^ cells were seeded in 24-well plates with the same medium at 37°C, 5% CO_2,_ and high humidity. When cells reached 70% confluence, they were washed with DMEM High glucose (without FBS and gentamicin) and subjected to treatment.

The GFP expression and cytoplasmic membrane integrity were performed in four experimental groups: DNA 1 (37.5 µg/mL of pmhyGENIE-5); DNA 2 (150 µg/mL of pmhyGENIE-5); polyplex 1 and polyplex 2. The control group consisted of cells not exposed to eDNA or polyplex; it was analyzed only for cytoplasmic membrane integrity. After the incubation period, the medium was replaced with DMEM + 10% of FBS + 50 IU/mL gentamicin, and the cells were incubated for an additional 72 h. All transfections were performed in quintuplicate. The experimental design is shown in Figure 1C.

Cells were scrapped, centrifuged (700 x g per 5 min), and resuspended in DMEM + 10% of FBS (100 µL) for the evaluations. Cell samples were analyzed using flow cytometry as described in the previous experiment. GFP expression was evaluated on the FL1 channel (FL1: 533 ± 30 nm). Cytoplasmic membrane integrity was assessed with Propidium Iodide (PI; 50 μg/mL; BP: 610/20 nm). After staining, PFF samples were incubated at 37°C for 10 min in the dark before flow cytometry analysis.

### Evaluation of polyplex cytotoxicity

For this evaluation, 2x105 cells were seeded in 24-well plates with DMEM + 10% FBS + 50 IU/mL gentamicin at 37°C, 5% CO2, and high humidity. When cells reached 70% confluence, they were washed with DMEM High glucose (without FBS and gentamicin) and subjected to treatment. The assessments were performed in three experimental groups: the Control group (cells not exposed to polyplex), polyplex 1, and polyplex 2. All transfections were performed in quintuplicate. The experimental design is shown in Figure 1C.

The PEI cytotoxicity potential was evaluated using the LIVE/DEAD® Viability/ Cytotoxicity Assay kit for mammalian cells (Invitrogen, Waltham, MA, USA). Fibroblast cells were cultured and transfected as previously described in item 2.5. Cells were exposed to polyplexes (1 and 2) previously diluted in a culture medium for 30 min. After that, cells were cultured in DMEM + 10% of FBS + 50 IU/mL gentamicin for 24 h, 48 h, and 72 h at 37°C and under 5% CO_2_. The Control group consisted of cells not exposed to PEI.

The test was performed after washing every well with PBS and carried out using the LIVE/DEAD® Viability/Cytotoxicity Assay kit for mammalian cells (Invitrogen, Waltham, MA, USA). In brief, the calcein green AM ester and ethidium homodimer were mixed and added to wells at room temperature and incubated for 40 minutes. The dish was kept in the dark at 37 °C for 30 min. The cells were analyzed using a flow cytometer (Accuri C6plus, Becton-Dickinson, USA) in 24, 48, and 72 hours of exposure; then, the percentage of cell viability per group was determined. The rate of live cells was calculated from the fluorescence readings defined according to the kit instructions.

### Statistical analysis

All data evaluated by MedCalc©. Independent variables were considered: PEI-FITC concentration, time, and transfection methods (DNA or Polyplex). Dependent variables were considered the percentages of internalization of PEI-FITC, cells expressing GFP, cells with damage to the cytoplasmic membrane, and viable cells. The Shapiro-Wilk test assessed the normal distribution. Comparisons between independent variables and the interactions were performed using a two-way analysis of variance for ANOVA, with Bonferroni as a post-test. When data were not normally distributed, the Kruskal-Wallis test was used with Dunn as a post-test. When applied, the Spearman test was used as a correlation test.

## Results

### Experiment 1: Optimization of PEI concentration and incubation time

No interaction between an incubation time and PEI-FITC concentration was observed (P=0.989). Furthermore, the input of PEI-FITC was not affected by the incubation time, with no difference observed between the averages of the percentages of cells labeled by FITC between the times (50.94 ± 1.06; 51.10 ± 1.06; 52.29 ± 1.06; respectively for 30 min, 1 and 2 hours; P = 0.620).

**Figure 2:**
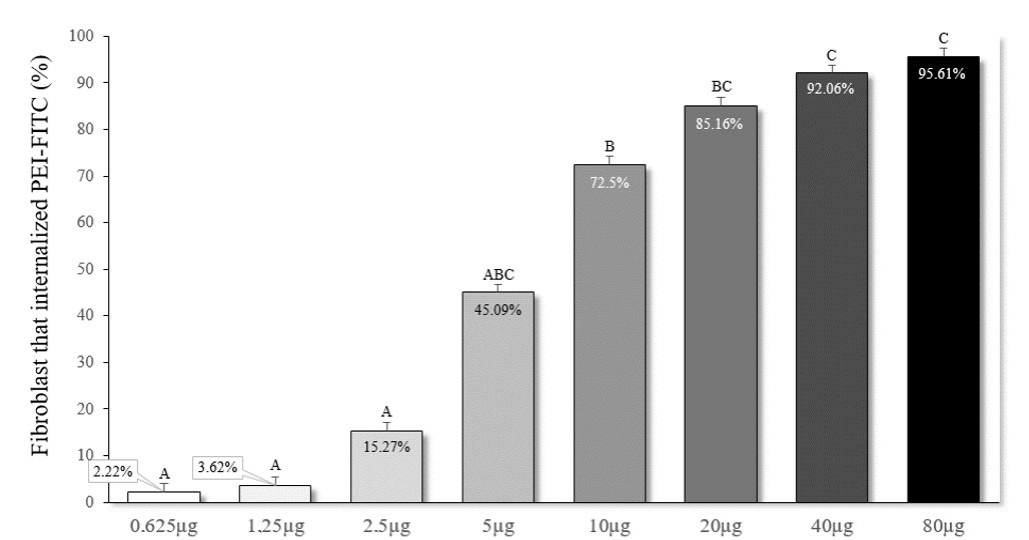
Internalization of PEI-FITC in porcine fetal fibroblasts submitted to different concentrations of PEI-FITC. Data represent percentage averages ± standard error of the mean (SEM). Different letters indicate significant differences between groups (P <0.05) (Marques, 2024).

On the other hand, a high correlation was verified between the percentage of PFF labeled with FITC and the concentration of PEI-FITC used, presenting a correlation coefficient (r) of 0.974 (P ≤ 0.0001). Also, PFFs treated with PEI-FITC at 80, 40, and 20 µg/mL showed higher internalization rates; the 5, 10, and 20 µg/mL concentrations presented intermediary rates and 0.625; 1.25 and 2,5 µg/mL concentrations showed low rates (Figure 2). Based on these results, 10 and 40 µg/mL concentrations were selected for the production of the polyplexes, representing concentrations that present intermediary and high internalization of PEI-FITC, respectively.

### Experiment 2: Efficiency of polyplexes in the production of PFFs expressing GFP

PEI micelles and pmhyGENIE-5 presented, respectively, 20 ± 1 and 135 ± 1 nm of hydrodynamic diameter, and zeta potential measurements were 2 ± 1 and -33±2 mV. Polyplexes containing an N/P ratio of 2 resulted in 131 ± 1 nm and a zeta potential of -14 ± 1 mV.

#### GFP expression

There was no interaction between the concentration of DNA (37.5 µg/mL or 150 µg/mL of pmhyGENIE-5) or polyplex (10 or 40 µg/mL) and the evaluation times (24, 48, or 72 h) in terms of percentage of cells expressing GFP (P = 0.666), because of the results of the concentration and time were described separately.

**Figure 3:**
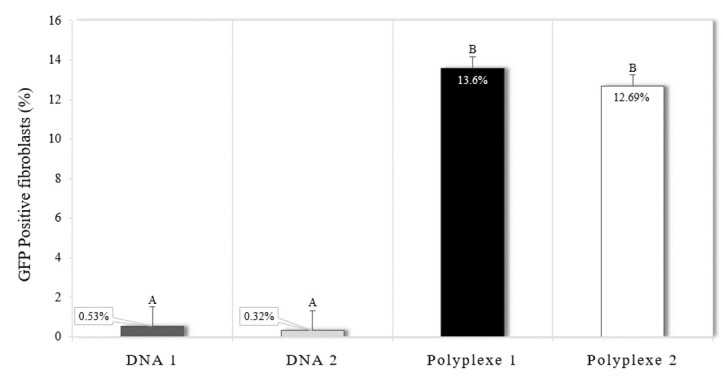
Expression of green fluorescent protein in porcine fetal fibroblasts after incubation with DNA and the polyplexes, being DNA 1 (37.5 µg/mL of pmhyGENIE-5); DNA 2 (150 µg/mL of pmhyGENIE-5), polyplex 1 (10 µg/mL of PEI and 37.5 µg/mL of pmhyGENIE-5) and polyplex 2 (40 µg/mL of PEI and 150 µg/mL of pmhyGENIE-5). Data represent percentage averages ± standard error of the mean (SEM). Different letters indicate significant differences between groups (P <0.05).

**Figure 4:**
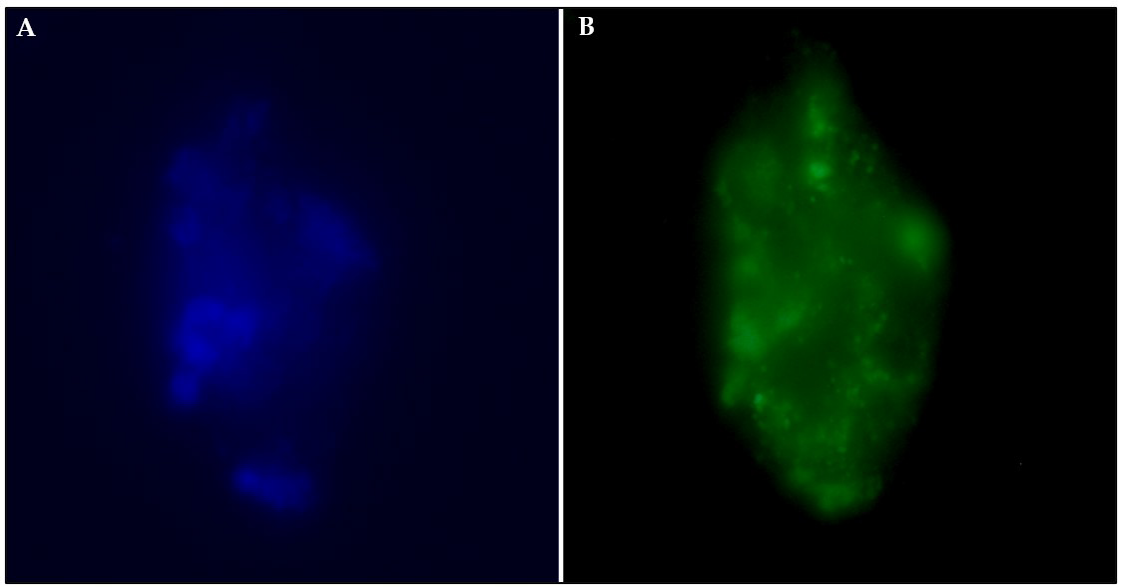
Fluorescence microscopy images of GFP expression in transfected porcine fetal fibroblasts in suspension. Porcine fetal fibroblasts were transfected with the polyplex 1 (10 µg/mL; 30 min), and images were collected 72 h after transfection. A: Porcine fetal fibroblasts are labeled with a blue, fluorescent dye specific for the nucleus (Hoechst 333422). B: Expression of green fluorescent protein in porcine fetal fibroblasts. Cells were examined on slides (Axio Observer A1, Carl Zeiss AG, Oberkochen, Germany). GFP and Hoesch fluorescence were detected in the green (470-490 nm) and blue (355–465 nm) channels, respectively.

Transfection using polyplexes 1 and 2 had similar expression rates of the fluorescent protein (13.60% ± 0.97 and 12.69% ± 0.97, respectively; P = 0.872; Figures 2 and 3). Incubation of cells with only DNA, without the presence of PEI, showed low rates of GFP expression (0.53% ± 0.97 and 0.32% ± 0.97 for DNA 1 and 2, respectively), not differing between and differing from the two groups in which PEI was used (P < 0.001) (Figures 3 and 4). Additionally, since the control group would not produce genetically modified PFFs, the GFP expression was not evaluated for this experimental group.

Besides, there was no difference in the percentage of cells expressing GFP in the evaluated periods (7.43 ± 0.69%, 6.75 ± 0.69%, and 6.17 ± 0.69% for the 24, 48, and 72 h after transfection, respectively).

### Evaluation of cytoplasmic membrane integrity

There was no interaction between the concentration of DNA (37.5 µg/mL or 150 µg/mL of pmhyGENIE-5) or polyplex (10 or 40 µg/mL) or Control and the evaluation times (24, 48, or 72 h) in terms of percentage of cells with cytoplasmic membrane damage (P = 0.744), because of the results of the concentration and time were described separately.

**Figure 5:**
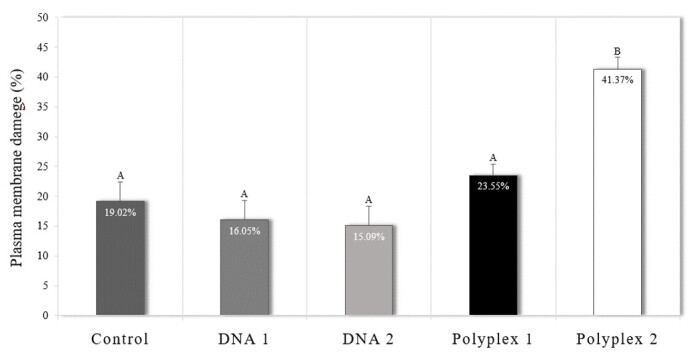
Damage rates of the cytoplasmic membrane in porcine fetal fibroblasts after incubation with DNA and the polyplexes, being DNA 1 (37.5 µg/mL of pmhyGENIE-5); DNA 2 (150 µg/mL of pmhyGENIE-5), polyplex 1 (10 µg/mL of PEI and 37.5 µg/mL of pmhyGENIE-5) and polyplex 2 (40 µg/mL of PEI and 150 µg/mL of pmhyGENIE-5). Data represent percentage averages ± standard error of the mean (SEM). Different letters indicate significant differences between groups (P <0.05).

When investigating the cytoplasmic membrane damage caused by different concentrations of DNA (37.5 or 150 µg/mL of pmhyGENIE-5) or polyplex (10 or 40 µg/mL), it was observed that PFF that were incubated with polyplex 2 had higher rates of cytoplasmic membrane injury than all other groups (P ≤ 0.0001). The different groups did not differ, nor did the control group (Figure 5).

Furthermore, there was no difference in the percentage of cells with cytoplasmic membrane damage in the evaluated periods (21.02 ± 2.17%, 21.19 ± 2.14%, and 26.94 ± 2.14% for the 24, 48, and 72 h after transfection, respectively).

### Evaluation of polyplex cytotoxicity

**Figure 6:**
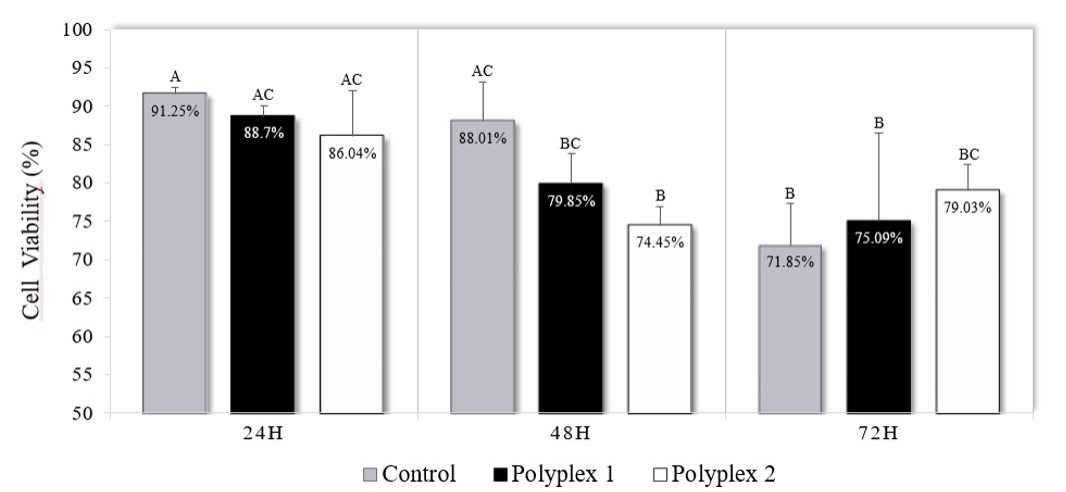
Cell viability assay in porcine fetal fibroblasts after transfection with polyplex 1 (10 µg/mL of PEI and 37.5 µg/mL of pmhyGENIE-5) and polyplex 2 (40 µg/mL of PEI and 150 µg/mL of pmhyGENIE-5). Data represent percentage averages ± standard error of the mean (SEM). Different letters indicate significant differences between groups (P <0.05).

There was an interaction between polyplex and post-transfection evaluation periods (24, 48, or 72 h) for the percentage of living cells (P = 0.006). All groups were observed to always have high cell viability rates. Furthermore, it was observed that at 24 hours, there was no difference between the groups. In the 48 hours, polyplex 2 presented inferior results to the Control, and after 72 hours of culture, all groups showed results below the 24-hour rates, with no difference between them (Figure 6).

## Discussion

In this work, we have optimized the conditions for transfecting PFF using PEI as a carrier. To investigate the time required for their internalization, PEI was derivatized with FITC so that the fluorescence measured in the cells could be a marker of internalization. It was possible to verify that the incubation time did not affect the internalization of PEI-FITC, so 30 minutes was enough for complex uptake. Our results corroborate with other studies, in which no difference was observed for PEI-FITC internalization by porcine female fibroblasts after 3 or 6 h (Souza et al., 2018). Silva et al. (2018) also observed that incubation for 10 min or 2 h did not differ in internalization by porcine sperm cells. When evaluating the mechanism of endocytosis in mouse fibroblasts (lineage L929) using Branched PEI (25 kDa) (Rémy-Kristensen et al., 2001) demonstrated that the polyplexes were efficiently absorbed in less than 10 min in endosomes that did not exceed 200 nm in diameter. In another study with branched PEI (25 kDa), it was demonstrated that 30 minutes after transfection, the PEI/pDNA complexes started to bind to cell surfaces and form aggregates (Godbey et al., 1999).

Unlike incubation time, the concentration of polymer affected the internalization of PEI-FITC. Although all concentrations of PEI-FITC were internalized in PFF, we observed that the percentage of FITC+ cells increased proportionally according to the concentration of PEI. Although Silva et al. (2018) have observed no differences in the rate of porcine sperm cells that internalized PEI-FITC at concentrations ranging from 0.5 – 4 mg/mL, suggesting the concentrations used were possibly higher than the required. In this study, our results showed that with concentrations above 10 µg/mL of PEI, it was possible to reach a large number of marked PFF (72.5%). The 40 and 80 µg/mL concentrations provided a similar percentage of PFF marked PEI-FITC (more than 90% of the cells), suggesting that 40 µg/mL of PEI is already enough to reach the maximum of marked PFF under the conditions evaluated. Based on that, 10 and 40 µg/mL concentrations were chosen to transfect the PFF. Although PFF could be easily obtained and cultured, a high transfection rate is desired. It would positively impact the establishment of transgenic strains since screening and selecting the population of transgenic cells is laborious.

The studies to optimize concentration and incubation time led to the following conditions: two polyplex compositions (10 and 40 µg/mL of PEI) with an N/P ratio of 2 and an incubation time of 30 minutes. Thus, the PFF were transfected using these two polyplexes protocols, and GFP expression and plasma membrane integrity were assessed over three days. Both polyplexes could transfect the fetal fibroblasts, and approximately 12% of fibroblasts expressed the green fluorescent protein. Different rates of GFP expression are reported using PEI protocols, but most of the expressions are transient. A gradual drop is observed in the percentage of cells that expressed GFP due to the non-integration of the gene of interest into the host cell's genetic material.

The percentage of GFP-positive fibroblasts did not differ over the three-day post-transfection assessment in our study, suggesting that the plasmid reached the nucleus and possibly was integrated into the genome, just as the GFP gene was transcribed and translated into green protein fluorescent. Coonrod and Horwitz 1997 (Coonrod et al., 1997) observed that the DNA's integrity that reaches the nucleus seems to be a determinant of gene transfer efficiency. The degradation of nucleic acids by cytoplasmic nucleases during cell internalization or transit to the nucleus and the fusion of endosomes containing exogenous DNA with lysosomes is decisive for transfection (Wattiaux et al., 2000; Sanz et al., 2012). In this respect, the “electron sponge” effect proposed for PEI could be advantageous since pDNA receives additional protection against degradation.

Another possible explanation for the stability of the transfection may be related to the nature of the plasmid. In our work, the used plasmid is the vector with integration based on the piggyBac transposon. Transposons are mobile genetic elements that act through a “cut and paste” transposition mechanism through the transposase enzyme. Transposases recognize and bind to transposons flanking inverted repeating elements, cut this segment of donor DNA, and reinsert it into the recipient genome. These properties have proven invaluable for delivering genes in applications such as transgenesis, mutagenesis for cancer research, or gene therapy. Marh et al. (2012) demonstrated a five-fold greater efficiency in producing genetically modified mice when using the piggyBac vector to perform pronuclear microinjection. In this sense, the PEI lysosomal escape mechanism combined with the plasmid nature may be related to the stability of GFP expressions, as shown in this work.

A significant disadvantage of using PEI as a transfection reagent is the cytotoxic effect on exposed cells and tissues. Studies show that PEI presents two types of cytotoxicity, an immediate one correlated with membrane damage and a delayed one related to the decomplexation of the PEI inside the cell (Godbey, Wu and Mikos, 1999. Thus, we investigated the effect of PEI concentration on cytoplasmic membrane integrity and cell viability at different periods after transfection.

We observed that the PEI concentration affected the integrity of the cytoplasmic membrane. The 10 µg/ml concentration did not cause significant damage to the cell membrane. On the other hand, 40 µg/ml concentration led to a higher damage rate to the cytoplasmic membrane. Fischer et al. (1999) suggested that PEI/pDNA complexes form aggregates on the surface of the plasma membrane, destabilizing it and resulting in cell disruption.

A commonly reported finding is impaired cell viability after polymer transfections (Forcato et al., 2017). In our study, the PEI concentration did not affect cell viability. Both polyplexes showed similar percentages of live cells post-transfection. At the end of 72 hours of culture, the cell viability of all groups was greater than 70%. The low cytotoxicity could be related to the incubation time, which was only 30 minutes. Luo et al. (2015) demonstrated that the 30-minute incubation generated minimal cytotoxicity in PEI-mediated transfection.

The high viability of the transfected PFF may also be associated with the zeta potential values of our polyplexes. The zeta potential of the polyplexes was -14 mV, a value lower than the plasmid ZP (-33.1 mV), demonstrating a reduction in charges. However, there was insufficient free PEI to interact with cellular components and impair their functions. Although this benefit has not yet been proven, many studies report the importance of free PEI for efficient transfection. Furthermore, free PEI can compete with polyplexes for binding sites on the cell surface, thus reducing cell uptake.

## Conclusion

Further studies should be conducted to optimize an optimal polymer/pDNA ratio for porcine fetal fibroblasts, ensuring that no pDNA or polymer remains free. PEI concentrations between 10 – 80 µg/ml promote high incorporation rates, even in periods as short as 30 minutes. Furthermore, it can be stated that the transfection condition used in Polyplexes 1 (10 µg/mL of PEI and 37.5 µg/mL of pmhyGENIE-5 for 30 min) efficiently produces genetically edited porcine fetal fibroblasts with low cell damage.

## Data Availability

Data sharing is not applicable.
